# Effect of a law amendment on dosimeter wearing in medical radiation workers: observational study

**DOI:** 10.1186/s13244-026-02218-3

**Published:** 2026-02-10

**Authors:** Satoru Matsuzaki, Koichi Nakagami, Tomoko Kuriyama, Koichi Morota, Go Hitomi, Hiroko Kitamura, Takashi Moritake

**Affiliations:** 1 Department of Radiation Regulatory Science Research, Institute for Radiological Science, National Institutes for Quantum Science and Technology (QST), Chiba, Japan; 2 Department of Radiology, Wakamatsu Hospital of the University of Occupational and Environmental Health, Japan, Kitakyushu, Japan; 3 Department of Occupational and Community Health Nursing, School of Health Sciences, University of Occupational and Environmental Health, Japan, Kitakyushu, Japan; 4 Department of Environmental Health, National Institute of Public Health, Wako, Japan; 5 Department of Radiological Technology, Kawasaki Medical School Hospital, Kurashiki, Japan

**Keywords:** Dosimeter wearing rate, Medical radiation workers, Visual compliance survey, Law amendment, Radiation protection

## Abstract

**Objectives:**

To evaluate the impact of a law amendment that reduced the eye lens dose limit on the use of personal dosimeters among radiation workers in medical settings.

**Materials and methods:**

A repeated cross-sectional survey was conducted at medical institutions across three periods: before the law amendment (control) and during the promulgation and implementation periods. Surveyors (radiological technologists) at each participating medical institution recorded dosimeter-wearing status among radiation workers. Data were collected via mail or email and analysed. The observed workers included physicians, nurses, and radiological technologists.

**Results:**

The surveys were collected from 1194 workers in the control period, 1374 in the promulgation period, and 1194 in the implementation period, totalling 3762 workers. Post-law amendment, the overall wearing rate of primary personal dosimeters significantly increased from 64.6% to 77.9% (*p* < 0.001). Significant increases in wearing rates were observed among physicians and radiological technologists (*p* < 0.001). Among occupations, physicians showed the lowest wearing rates across all periods (control: 35.8%, promulgation: 56.7%, implementation: 62.6%), whereas radiological technologists showed the highest (control: 92.7%, promulgation: 98.5%, implementation: 99.5%). Regarding physician specialities, orthopaedic surgery exhibited the lowest compliance (control: 11.3%, promulgation: 35.4%, implementation: 24.7%). The proportion of workers without provision of a personal dosimeter declined from 5.9% to 1.9% (*p* < 0.001).

**Conclusions:**

Despite overall improvement following the law amendment, low compliance among physicians, particularly in orthopaedics, indicates the need for targeted interventions.

**Critical relevance statement:**

Although dosimeter-wearing rates improved after Japan’s eye dose limit revision, persistent low physician compliance—especially in orthopaedics—highlights the need for targeted strategies to strengthen radiation protection in clinical practice.

**Key Points:**

- The effect of reduced eye dose limits on dosimeter use remains unclear.
- Personal dosimeter usage increased significantly after the law amendment. Compliance remained low among orthopaedic physicians despite regulatory tightening.
- Targeted interventions are needed for low-compliance groups to ensure radiation protection.

**Graphical Abstract:**

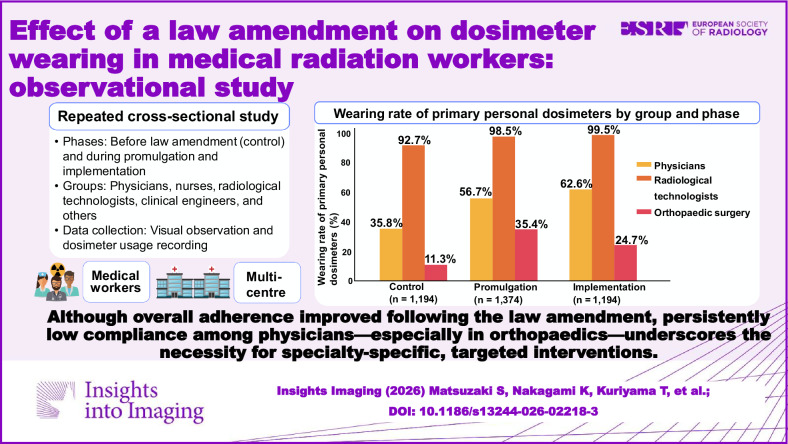

## Introduction

The International Commission on Radiological Protection recommends that the equivalent dose limit for the eye lens should not exceed 20 mSv/year on average over 5 years and 50 mSv in any single year [1], prompting global law amendments. In Japan, an amendment to the Ordinance on Prevention of Ionizing Radiation Hazards (No. 41, 1972) [2] (hereafter, “Ionizing Radiation Ordinance”), promulgated on 1 April 2020 and implemented in April 2021, led to a new equivalent dose limit for the eye lens, reduced from the previous limit of 150 mSv/year. Additionally, activities to raise awareness, including distributing posters and notifications, were conducted to ensure that medical radiation workers wear personal dosimeters in Japan.

Article 8 of the Ionizing Radiation Ordinance mandates that employers ensure their medical radiation workers wear personal dosimeters and conduct dose measurements [2]. Nevertheless, previous studies have highlighted non-compliance issues, for example, wearing rates range 0–93.5% among physicians [3–22], 39.7–94% among nurses [7, 20–23], and 24–98.7% among radiological technologists [20–26] (Supplementary Table 1). Furthermore, the proportion of Japanese physicians wearing dosimeters very frequently (defined as ≥ 80% of the time) varied by speciality: 90% in radiology, 36% in gastrointestinal internal medicine, and 45% in orthopaedic surgery [27]. Moreover, Abdelrahman et al noted potential social desirability bias in such data, raising concerns about the accuracy of self-reported dosimeter usage [15]. Ko et al found a 2.39 mSv discrepancy between National Dosimetry Registry data and actual doses measured by properly worn active dosimeters for workers wearing dosimeters less than 75% of the time [28]. Therefore, dosimeter usage needs to be accurately evaluated to ensure data reliability.

Non-compliance with wearing personal dosimeters stems from the failure of hospital employers to provide dosimeters (e.g. no provision of personal dosimeters [5, 21, 26, 27, 29–32] and insufficient education on radiation protection [4, 8, 11, 14, 16, 19, 21, 22, 27–29, 31–35]) and the failure of medical radiation workers to wear them (e.g. failure to remember [5, 6, 11, 21, 26, 35, 36], culture of radiation protection practice [16, 19, 32, 35], work restrictions due to dose limits [10, 30, 35, 36]). Among these, ‘work restrictions due to dose limits’ may cause both workers who are unaware of their exposure levels and those with high exposure levels to fear exceeding the limits and prevent them from wearing dosimeters. Furthermore, medical radiation workers may rationalise non-compliance based on inadequate knowledge or to prioritise patient care [32, 37]. Hence, the hypothesis for this study was that concerns over surpassing the newly lowered dose limit due to the law amendment might further reduce the wearing rate of personal dosimeters.

This study aimed to determine the impact of the amendment to the Ionizing Radiation Ordinance, which lowered the equivalent dose limit for the eye lens, on the use of personal dosimeters by medical radiation workers. Accordingly, radiological technologists at medical institutions across Japan observed and documented the dosimeter-wearing status of medical radiation workers at their facilities during three periods: before announcement of the law amendment (control period), before enforcement (promulgation period), and after enforcement (implementation period). By analysing these observations over time, the study evaluated changes in dosimeter compliance in response to the regulatory change. Additionally, the distribution of personal dosimeters for medical radiation workers was surveyed during three distinct periods.

## Materials and methods

### Ethical approval

This study was approved by the Ethics Committee of the University of Occupational and Environmental Health, Japan (approval number: R1-053). The study involved visual observation of medical radiation workers in their usual clinical environment by trained surveyors affiliated with each participating institution. Informed consent was obtained in writing from all surveyors after providing a comprehensive explanation of the study objectives and procedures. For observed workers, individual consent was waived by the ethics committee in accordance with national guidelines because the data collected were from non-intrusive, anonymised behavioural observation without intervention. All procedures adhered to the Declaration of Helsinki and relevant institutional and national ethical standards.

### Regulatory requirements for dosimeter use in Japan

In Japan, the Ionizing Radiation Ordinance requires that radiation exposure doses be measured for workers employed in designated controlled areas. Article 8 stipulates that personal dosimeters be worn on the chest for men, or for women diagnosed as unable to become pregnant, and on the abdomen for others (standard position). These dosimeters are typically worn under the lead apron. Additionally, when the most highly exposed body part is the head/neck, chest/upper arms, or abdomen/thighs, an additional dosimeter should be worn at that location, except when already covered. If another site is expected to receive the highest exposure, a dosimeter must also be placed accordingly. Notably, if a physician is not wearing a lead apron, the equivalent dose to the eye lens is calculated using the reading of the personal dosimeter worn in the standard position, whereas if a lead apron is worn, it is calculated using the reading of the additional personal dosimeter worn outside the lead apron.

### Study design

A repeated cross-sectional observational survey was conducted to evaluate the distribution and usage of personal dosimeters among radiation workers in radiation-controlled areas at medical institutions in Japan. Data were collected on voluntary dates chosen by each surveyor within the defined periods, which corresponded to the control, promulgation, and implementation phases of the law amendment. The control period, from 27 January to 31 March 2020, covered the time before the law’s promulgation, during which the distribution and wearing status of personal dosimeters were surveyed at the medical institutions. Similarly, the promulgation period was set from 1 April to 9 May 2020 and from 1 January to 31 March 2021, covering the period after the promulgation and notification of the law amendment. As the promulgation period coincided with the outbreak of novel coronavirus in Japan, the survey was conducted over two periods to account for a potential decrease in the observed workers. The implementation period, from 1 January to 31 March 2022, covered the time after the law’s implementation.

### Data collection method and content

The surveyors were radiological technologists regularly involved in radiological procedures at their institutions and familiar with the occupations of the staff observed, including physicians’ medical specialities. Surveyors downloaded the survey sheet from the designated website to record observations. Each surveyor visually examined the wearing status of personal dosimeters among medical radiation workers for each procedure in their facility’s radiation-controlled areas, such as the angiography and operating rooms. Personal dosimeter wearing status was visually inspected before, during, or immediately after the radiological procedures, with the results recorded on a survey sheet. All surveyors followed the same simple visual criterion: personal dosimeter use was recorded as “yes” if at least one personal dosimeter was visibly worn at the designated body sites, and as “no” if no dosimeter was visible. The key point is that transparency and fairness were ensured in this manner by having a third party conduct the survey confidentially. The procedure referred to the interventional procedures and diagnostics observed at different medical institutions. Therefore, these were not standardised across all sites, but rather reflected the specific practices commonly performed at each institution.

Collected items included: (1) observation date, (2) occupation of observed worker, (3) medical specialty (for physicians), and (4) distribution and wearing status of personal dosimeters. A detailed list of physician specialties is provided in Supplementary Table 2. Here, the personal dosimeters included were those legally required in Japanese medical institutions, namely, passive-type radio-photoluminescence glass, optically stimulated luminescence, and active-type electronic pocket dosimeters.

Notably, we did not collect information on the type, brand, or manufacturer of personal dosimeters, nor on the sex or age of the observed workers. Surveyors completed all items in a multiple-choice format and submitted the data via mail or email, which were then compiled into an analysis database.

### Recruitment of surveyors

Surveyors were publicly recruited from among radiological technologists nationwide via the website of the Department of Occupational and Community Health Nursing, School of Health Sciences, University of Occupational and Environmental Health, Japan. Recruitment was also conducted individually by the authors and through relevant academic societies to which radiological technologists belong (such as the Japan Association of Radiological Technologists).

### Outcome measures

Three key measures related to the use of personal dosimeters were defined: primary dosimeter-wearing rate, additional dosimeter-wearing rate, and distribution rate. The primary dosimeter refers to the personal dosimeter worn at the standard position specified in the Ionizing Radiation Ordinance, typically under the lead apron on the chest or the abdomen. The primary dosimeter-wearing rate was defined as the proportion of workers wearing this dosimeter at the appropriate position among all observed workers, and was calculated using Eq. (1):

1 $$	{{{\mathrm{Primary}}}}\; {{{\rm{dosimeter}}}} {\mbox{-}} {{{\mathrm{wearing}}}}\; {{{\rm{rate}}}} \\ 	 \quad = ({{{\mathrm{Number}}}}\; {{{\rm{of}}}}\; {{{\rm{workers}}}}\; {{{\rm{wearing}}}}\; {{{\rm{primary}}}}\; {{{\rm{dosimeter}}}})/ \\ 	 \qquad ({{{\mathrm{Total}}}}\; {{{\rm{number}}}}\; {{{\rm{of}}}}\; {{{\rm{observed}}}}\; {{{\rm{workers}}}})$$

The additional dosimeter refers to an extra dosimeter worn over the lead apron at another body site when that site is expected to receive the highest radiation exposure, as required under certain conditions by the Ordinance. The additional dosimeter-wearing rate was defined as the proportion of workers wearing this dosimeter among all observed workers, and was calculated using Eq. (2):

2 $$	 {{{\mathrm{Additional}}}}\; {{{\rm{dosimeter}}}} {\mbox{-}} {{{\mathrm{wearing}}}}\; {{{\rm{rate}}}} \\ 	 \quad = ({{{\mathrm{Number}}}}\; {{{\rm{of}}}}\; {{{\rm{workers}}}}\; {{{\rm{wearing}}}}\; {{{\rm{additional}}}}\; {{{\rm{dosimeter}}}}) / \\ 	 \qquad ({{{\mathrm{Total}}}}\; {{{\rm{number}}}}\; {{{\rm{of}}}}\; {{{\rm{observed}}}}\; {{{\rm{workers}}}})$$

The distribution rate was defined as the proportion of workers to whom a dosimeter had been provided, regardless of whether they were wearing it correctly, and was calculated using Eq. (3):

3 $${{{\mathrm{Distribution}}}}\; {{{\rm{rate}}}} = 	 \, ({{{\mathrm{Number}}}}\; {{{\rm{of}}}}\; {{{\rm{workers}}}}\; {{{\rm{provided}}}}\; {{{\rm{with}}}}\; {{{\rm{dosimeter}}}}) / \\ 	({{{\mathrm{Total}}}}\; {{{\rm{number}}}}\; {{{\rm{of}}}}\; {{{\rm{observed}}}}\; {{{\rm{workers}}}})$$

These rates were calculated separately for each of the three defined survey periods: control, promulgation, and implementation. Wearing rates were further compared by occupation, and for primary dosimeters, also by medical specialty.

## Statistical analyses

To evaluate temporal changes in dosimeter-wearing and distribution rates, Fisher’s exact test was applied for comparison across the three periods (control, promulgation, and implementation), with Bonferroni correction for multiple comparisons. To assess the association between dosimeter-wearing behaviour and occupation or medical specialty, Firth’s logistic regression was performed, with dosimeter wearing (yes/no) as the outcome and occupation and medical specialty as predictors. Odds ratios and 95% confidence intervals were estimated. All analyses were performed using SPSS version 30 (IBM Corp.), including functions available through the SPSS Extension Hub. Significance was set at *p* < 0.05.

## Results

### Worker and surveyor characteristics

**Fig. 1:**
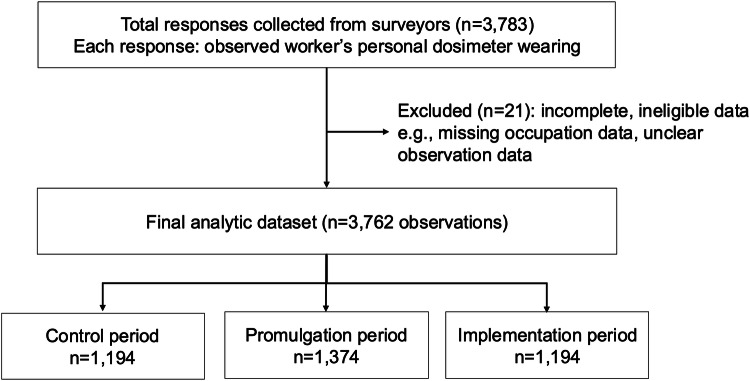
Repeated cross-sectional observational survey (three periods) conducted in medical institutions across Japan

During the control, promulgation, and implementation periods, 1206, 1378, and 1199 observed workers were recorded (Fig. 1). Owing to documentation errors, 12, 4, and 5 records from each period were excluded. In the final analysis, data were collected from 1194 workers in the control period, 1374 in the promulgation period, and 1194 in the implementation period, totalling 3762 workers (Fig. 1).

In the control period, 76 surveyors at 38 medical institutions participated in the observation. In the promulgation and implementation periods, 53 surveyors at 29 medical institutions, and 36 surveyors at 23 medical institutions conducted the same observation, respectively. Across the three survey periods, 165 (counting duplicates as one: 115) radiological technologists participated as surveyors: 77 (67.0%), 26 (22.6%), and 12 (10.4%) in one, two, and three periods, respectively (Table 1).

**Table 1:** Breakdown of surveyors by survey period

Survey period	No. of surveyors
Control period	76
Promulgation period	53
Implementation period	36
Total (count duplicates as a single person)	165 (115)
One time participation	
Control period	41 (35.7%)
Promulgation period	20 (17.4%)
Implementation period	16 (13.9%)
Total	77 (67.0%)
Two times participation	
Control and promulgation periods	18 (15.7%)
Control and implementation periods	5 (4.3%)
Promulgation and implementation periods	3 (2.6%)
Total	26 (22.6%)
Three times participation	
Total	12 (10.4%)

### Primary personal dosimeter-wearing rate by occupation and medical speciality

Table 2 shows the changes in the wearing rate of primary personal dosimeters for each occupation. Post-law amendment, the overall wearing rate of primary personal dosimeters increased significantly from 64.6% to 77.9% (*p* < 0.001). Physicians, radiological technologists, and clinical engineers experienced significant increases in wearing rates from the control to promulgation and implementation periods (*p* < 0.001 for all). A weak association was observed between the law amendment and the wearing rates of primary personal dosimeters (Cramer’s V: 0.22 for physicians, 0.17 for radiology technicians and 0.25 for clinical engineers). Across all periods, physicians had the lowest wearing rates, whereas radiological technologists had the highest. Compared with radiological technologists, physicians exhibited odds ratios of 0.05 in the control period, 0.02 in the promulgation period, and 0.01 in the implementation period, all showing significant differences.

**Table 2:** Impact of the law amendment on primary personal dosimeter wearing rates by occupation

Occupation	Control period			Odds ratio (95% CI)	Promulgation period			Odds ratio (95% CI)	Implementation period			Odds ratio (95% CI)	*p* -value	Cramer’s measures	
	Total number	Number of dosimeter-wearing workers	(%)		Total number	Number of dosimeter-wearing workers	(%)		Total number	Number of dosimeter-wearing workers	(%)			Cramer’s V	*p* -value
Physicians	461	165	35.8	0.05 (0.03, 0.07)	573	325	56.7^*^	0.02 (0.01, 0.05)	479	300	62.6^*^	0.01 (0.001, 0.04)	< 0.001	0.22	< 0.001
Nurses	326	249	76.4	0.26 (0.16, 0.42)	379	285	75.2	0.05 (0.02, 0.12)	376	305	81.1	0.03 (0.003, 0.11)	0.12	0.06	0.12
Radiological technologists	313	290	92.7		270	266	98.5^*^		215	214	99.5^*^		< 0.001	0.17	< 0.001
Clinical engineers	82	64	78.0	0.28 (0.15, 0.55)	123	82	66.7	0.03 (0.01, 0.08)	85	78	91.8^*, **^	0.07 (0.01, 0.34)	< 0.001	0.25	< 0.001
Other	12	3	25.0	0.03 (0.01, 0.10)	29	17	58.6	0.02 (0.01, 0.07)	39	33	84.6^*, **^	0.04 (0.004, 0.18)	< 0.001	0.44	< 0.001
Total	1194	771	64.6		1374	975	71.0^*^		1194	930	77.9^*, **^		< 0.001	0.12	< 0.001

Odds ratios were determined with the values for radiological technologists serving as the reference

*CI* confidence interval

^*^ Indicates that the value is significantly different compared to that in the control period

^**^ Indicates that the value in the implementation period is significantly different compared to that in the promulgation period

Table 3 presents the changes in the wearing rate of primary personal dosimeters among physicians (control period: *n* = 461, promulgation period: *n* = 573, and implementation period: *n* = 479) by medical speciality. Significant increases were found for the observed workers from cardiology and gastroenterology departments from the control to implementation periods (*p* < 0.001 for both). Conversely, the wearing rate in orthopaedic surgery was 11.3% during the control period and increased slightly to 35.4% during the promulgation period; however, it decreased to 24.7% during the implementation period. The wearing rate for workers in orthopaedic surgery remained consistently low (odds ratio compared to radiology: 0.05 in the control period, 0.20 in the promulgation period, and 0.004 in the implementation period), whereas that in radiology was high across all periods. The observed workers from most medical departments showed significantly higher wearing rates during the promulgation and implementation periods than in the control period, with pulmonology and emergency medicine showing significant associations with the law amendment (Cramer’s V: 0.60 for pulmonology and 0.52 for emergency medicine).

**Table 3:** Effect of the law amendment on primary personal dosimeter usage among physicians by medical speciality

Specialised area	Control period			Odds ratio (95% CI)	Promulgation period			Odds ratio (95% CI)	Implementation period			Odds ratio (95% CI)	*p* -value	Cramer’s measures	
	Total number	Number of dosimeter-wearing workers	(%)		Total number	Number of dosimeter-wearing workers	(%)		Total number	Number of dosimeter-wearing workers	(%)			Cramer’s V	*p* -value
Cardiology	117	45	38.5	0.23 (0.10, 0.52)	194	123	63.4^*^	0.62 (0.31, 1.18)	103	70	68.0^*^	0.02 (0.0002, 0.17)	< 0.001	0.24	< 0.001
Gastroenterology	104	36	34.6	0.20 (0.08, 0.45)	75	52	69.3^*^	0.80 (0.36, 1.72)	74	53	71.6^*^	0.03 (0.0002, 0.20)	< 0.001	0.36	< 0.001
Orthopaedic Surgery	62	7	11.3	0.05 (0.02, 0.14)	48	17	35.4^*^	0.20 (0.08, 0.45)	85	21	24.7	0.004 (0.00003, 0.03)	0.01	0.22	0.01
Neurology	37	14	37.8	0.23 (0.08, 0.60)	40	13	32.5	0.18 (0.07, 0.42)	46	30	65.2^*, **^	0.02 (0.0002, 0.16)	0.005	0.30	0.005
Urology	36	15	41.7	0.27 (0.10, 0.71)	23	15	65.2	0.65 (0.23, 1.87)	36	21	58.3	0.01 (0.0001, 0.12)	0.17	0.20	0.17
Radiology	34	25	73.5		54	40	74.1		46	46	100^*, **^		< 0.001	0.33	< 0.001
Surgery	28	7	25.0	0.13 (0.04, 0.38)	45	19	42.2	0.26 (0.11, 0.60)	15	11	73.3^*^	0.03 (0.0002, 0.29)	0.01	0.33	0.01
Anaesthesiology	12	3	25.0	0.14 (0.03, 0.54)	20	1	5.0^*^	0.03 (0.003, 0.12)	32	15	46.9^**^	0.01 (0.0001, 0.08)	0.003	0.41	0.004
Emergency Medicine	11	4	36.4	0.22 (0.05, 0.87)	19	8	42.1	0.26 (0.09, 0.76)	10	10	100^*, **^	0.23 (0.001, 42.82)	0.003	0.52	0.004
Pulmonology	6	1	16.7	0.10 (0.01, 0.60)	10	10	100^*^	7.52 (0.87, 989.30)	16	9	56.3^**^	0.01 (0.0001, 0.13)	0.002	0.60	0.003
General Internal Medicine	4	2	50.0	0.37 (0.05, 2.73)	10	8	80.0	1.22 (0.29, 7.01)	5	4	80.0	0.03 (0.0002, 0.69)	0.64	0.28	0.64
Other	10	6	60.0	0.54 (0.13, 2.32)	35	19	54.3	0.42 (0.17, 1.02)	11	10	90.9	0.08 (0.001, 1.51)	0.09	0.29	0.10
Total	461	165	35.8		573	325	56.7^*^		479	300	62.6^*^		< 0.001	0.22	< 0.001

Odds ratios were determined with the values for radiology serving as the reference

*CI* confidence interval

^*^ Indicates that the value is significantly different compared to that in the control period

^**^ Indicates that the value in the implementation period is significantly different compared to that in the promulgation period

### Additional personal dosimeter-wearing rate by occupation

Table 4 shows the wearing rates of additional personal dosimeters by occupation. Throughout all periods, the overall wearing rate of additional personal dosimeters was lower than that of primary personal dosimeters, with the highest rate observed during the promulgation period (control period: 54.2%, promulgation period: 69.5%, implementation period: 68.9%). Physicians exhibited low wearing rates, whereas radiological technologists maintained high wearing rates across all three periods. These trends were similar to those for the wearing rates of primary personal dosimeters.

**Table 4:** Impact of the law amendment on additional personal dosimeter usage by occupation

Occupation	Control period			Odds ratio (95% CI)	Promulgation period			Odds ratio (95% CI)	Implementation period			Odds ratio (95% CI)	*p* -value	Cramer’s measures	
	Total number	Number of dosimeter-wearing workers	(%)		Total number	Number of dosimeter-wearing workers	(%)		Total number	Number of dosimeter-wearing workers	(%)			Cramer’s V	*p* -value
Physicians	229	73	31.9	0.22 (0.14, 0.34)	438	233	53.2^*^	0.04 (0.02, 0.09)	379	200	52.8^*^	0.03 (0.01, 0.08)	< 0.001	0.18	< 0.001
Nurses	143	104	72.7	1.22 (0.73, 2.04)	231	170	73.6	0.11 (0.05, 0.22)	238	168	70.6	0.07 (0.02, 0.17)	0.77	0.03	0.77
Radiological technologists	140	96	68.6		198	191	96.5^*^		157	153	97.5^*^		< 0.001	0.41	< 0.001
Clinical engineers	39	26	66.7	0.90 (0.43, 1.95)	85	65	76.5	0.13 (0.05, 0.29)	68	59	86.8^*^	0.18 (0.05, 0.56)	0.049	0.18	0.047
Other	1	0	0	0.15 (0.001, 2.94)	14	12	85.7	0.20 (0.05, 1.13)	3	2	66.7	0.05 (0.01, 0.61)	0.20	0.48	0.32
Total	552	299	54.2		966	671	69.5^*^		845	582	68.9^*^		< 0.001	0.13	< 0.001

Odds ratios were determined compared with the value of radiological technologists

*CI* confidence interval

^*^ Indicates that the value is significantly different compared to that in the control period

### Personal dosimeter distribution by occupation

Table 5 illustrates the changes in the distribution status of personal dosimeters. The proportion of workers without a personally allocated dosimeter significantly decreased from 5.9% in the control period to 4.6% in the promulgation period and 1.9% in the implementation period (*p* < 0.001). The proportion of workers allocated primary and additional personal dosimeters increased over the survey periods. Moreover, a weak association was observed between the distribution status of personal dosimeters and the transition to the law amendment.

**Table 5:** Impact of the law amendment on the distribution of personal dosimeters

Dosimeters provided	Control period		Promulgation period		Implementation period		*p*-value	Cramer’s measures	
	Total number	(%)	Total number	(%)	Total number	(%)		Cramer’s V	*p*-value
None	70	5.9	63	4.6	23	1.9^*, **^	< 0.001	0.17	< 0.001
Primary personal dosimeter only	572	47.9	345	25.1^*^	326	27.3^*^			
Both primary and additional personal dosimeters	552	46.2	966	70.3^*^	845	70.8^*^			
Total	1194		1374		1194				

^*^ Indicates that the value is significantly different compared to that in the control period

^**^ Indicates that the value in the implementation period is significantly different compared to that in the promulgation period

## Discussion

Our findings revealed an upward trend in the overall wearing rate of primary personal dosimeters following the law amendment. The wearing rate varied by occupation, with physicians exhibiting the lowest rates. Within the specialised areas of physicians, orthopaedic surgery showed low wearing rates across all periods. Despite differences between primary and additional personal dosimeters, some similarities were observed. Furthermore, the distribution status of personal dosimeters indicated a decrease in the number of workers not provided with a dosimeter, reflecting the impact of the law amendment.

Previous questionnaire-based studies have shown considerable variability in wearing rates across various occupations, with compliance seldom reaching 100% (Supplementary Table 1) [3–26, 38]. Our findings are broadly consistent with previous reports, which similarly showed that compliance remains suboptimal. However, many studies relied on self-reported questionnaires, which may contain social-desirability bias, as wearing dosimeters is generally desirable [15]. Kim et al found a discrepancy between self-assessed rates and true dosimeter-wearing rates, with a kappa coefficient of 0.39 for wearing personal dosimeters, indicating poor agreement [39]. As presented in Supplementary Table 1, previous studies on the personal dosimeter use relied on self-administered questionnaires, which may not accurately reflect the actual dosimeter-wearing status. Therefore, at this important milestone of the law amendment, the wearing rates of personal dosimeters need to be verified through independent third-party evaluations to ensure accurate dose evaluations for medical radiation workers. This study is the first globally to use a third-party visual survey to determine the true wearing rates of personal dosimeters and to evaluate changes in these rates among medical radiation workers in Japan during the promulgation and implementation of the law amendment.

The wearing rate of primary personal dosimeters tended to increase during the promulgation and implementation periods of the law amendment. The weak correlation suggests that the law amendment was not a negative factor but likely increased interest in dose measurements to avoid surpassing the newly lowered dose limit. Moreover, significant differences in the wearing rates of primary and additional personal dosimeters were observed across different occupations and medical specialities. These differences may arise from a silo mentality in specialised fields, where collaboration with other specialities is limited [40], as well as varying radiation protection cultures among occupations. In particular, orthopaedic surgeons had a low dosimeter-wearing rate despite being at high risk for radiation-induced cataracts and skin disorders [41, 42], suggesting a possible underestimation of previously reported exposure doses and highlighting the need for accurate measurement. Conversely, the increase in wearing rates among pulmonologists and emergency physicians was likely due to heightened interest in dose measurements following the law amendment. Furthermore, the similar trends in wearing rates between primary and additional personal dosimeters suggest that workers wear both when available, indicating an understanding of their importance.

Post-law amendment, the number of workers not provided with a personal dosimeter decreased. The current situation of appropriate exposure management by employers indicates the impact of the national law amendment and awareness-raising activities, such as distributing posters to promote dose measurements.

Our findings suggest that the wearing rate did not reach 100% even after the law amendment and awareness-raising activities, indicating the need for more intensive measures. For example, applying the Hawthorne effect, whereby attention to behaviour enhances performance, is important in encouraging medical radiation workers to wear personal dosimeters appropriately [32, 43, 44]. Specifically, implementing a system that improves the management of radiation safety culture is crucial [45]. This involves assigning supervisors to radiation workplaces, regularly auditing the wearing status of personal dosimeters, encouraging their use [46], and implementing educational programs. In Japan, a radiation management system based on ISO45001—an international standard for occupational safety and health management systems—has been established as a systematic, employer-led approach independent of human factors [47]. Advancing the audit system is expected to increase the wearing rate of personal dosimeters and enhance the efficiency of exposure management.

This study has some limitations. First, recall bias might have affected surveyors who recorded data after the completion of radiation treatment [17]. Second, although the survey results were derived from over 1000 cases in each period, the study used a sample survey, which may not accurately reflect the wearing rate of personal dosimeters in Japan. Therefore, the observed increases should be interpreted as group-level trends rather than individual-level improvements. Third, when the data were stratified based on physicians’ medical speciality, some subgroups included only a small number of physicians. This may have reduced the statistical power to detect differences in dosimeter-wearing behaviour. Therefore, even non-significant results in these subgroup analyses may underestimate meaningful differences in protective practices. Fourth, the limited evaluation period did not allow for capturing long-term changes. Fifth, although this study revealed increasing dosimeter-wearing trends and decreasing rates of workers without dosimeters over the three periods, the observed workers differed at each time point. Sixth, the observations were conducted by many radiological technologists from multiple institutions, with surveyor composition varying across the three periods. Although all surveyors followed a common simple visual criterion—marking “yes” if a dosimeter was visibly worn at specified sites and “no” if not—each worker–procedure combination was assessed by a single surveyor only, with no double-checking by multiple independent surveyors. Consequently, some misclassification due to overlooking or misjudging personal dosimeters cannot be ruled out, and dosimeter-wearing rates in certain specialities or periods may have been over- or underestimated. Despite these limitations, this study objectively clarified the status of distribution and wearing of personal dosimeters among medical radiation workers in Japan before and after the law amendment, marking an unprecedented and important achievement. Our findings provide a foundation for the future management of radiation safety culture and implementing improvements in exposure management.

## Conclusion

The law amendment and its notification raised interest in dose measurements, yielding an increase in the overall dosimeter-wearing rate and a decrease in the number of workers not provided with a dosimeter. However, the wearing rate did not reach 100%, and differences were observed across occupations and medical specialities, with significantly low rates among physicians and orthopaedic surgeons. Future efforts should include more intensive measures such as assigning managers, strengthening education, and introducing an audit system to manage radiation safety culture. This study is the first to clarify the impact of law reductions in radiation dose limits on the wearing rates of personal dosimeters, providing important insights for future radiation protection efforts.

## Supplementary information

ELECTRONIC SUPPLEMENTARY MATERIAL

## Data Availability

The datasets generated and analysed during the current study are not publicly available because the informed consent obtained from the participants did not include permission for unrestricted data sharing.
