# Supplementary material for: Effect of a law amendment on dosimeter wearing in medical radiation workers: observational study
Source: Insights Imaging. 2026 Feb 10;17:42. doi: 10.1186/s13244-026-02218-3 (PMC12891265; doi:10.1186/s13244-026-02218-3)
Supplement: Supplementary file 1 — ELECTRONIC SUPPLEMENTARY MATERIAL [file 13244_2026_2218_MOESM1_ESM.pdf]

Effect of a law amendment on dosimeter wearing in medical radiation workers: observational study
ELECTRONIC SUPPLEMENTARY MATERIAL

Supplementary Table 1 Wearing status of personal dosimeters in previous studies

References	Publication dates	Research methods	Locality	Personal dosimeter-use rate (%)			
				Physicians	Nurses	Radiology technicians	Others
Niklason [3]	1993	Questionnaire	N/A	40	N/A	N/A	N/A
Warren-Forward [33]	2008	Questionnaire	Australia	N/A	N/A	N/A	36*
Kim [4]	2010	Questionnaire	United States	52*	N/A	N/A	N/A
Elkoushy [5]	2011	Questionnaire	North America, Europe, Africa	34.3	N/A	N/A	N/A
Sánchez [6]	2012	Questionnaire	Spain	64	N/A	N/A	N/A
Bhatt [24]	2012	Questionnaire	Nepal	N/A	N/A	24	N/A
Vano [7]	2013	Questionnaire	Argentina	29.5	39.7	N/A	N/A
Shin [23]	2013	Questionnaire	South Korea	N/A	68.9		N/A
Söylemez [8]	2013	Questionnaire	Europe (Germany, France, Poland, Turkey, and the United Kingdom, among others)	28.2*	N/A	N/A	N/A
Dudley [9]	2015	Questionnaire	North America	35	N/A	N/A	N/A
Klein [10]	2015	Questionnaire	United States	71.4	N/A	N/A	N/A
Nugent [11]	2015	Questionnaire	Ireland	7.7*	N/A	N/A	N/A
Tunçer [12]	2017	Questionnaire	Turkey	5-10	N/A	N/A	N/A
Yun [25]	2017	Questionnaire	South Korea	N/A	N/A	66.5*–81.1*	N/A
Brun [13]	2018	Questionnaire	France	54.3–76	N/A	N/A	N/A
McCulloch [35]	2018	Questionnaire	United States	N/A	N/A	N/A	35*–64*
Joeris [14]	2018	Questionnaire	Africa, Asia, Europe, Latin America, the Middle East, and North America	21*	N/A	N/A	N/A
Abdelrahman [15]	2018	Questionnaire	Jordan	93.5	N/A	N/A	N/A
Whitney [16]	2019	Questionnaire	United States	25.7*	N/A	N/A	N/A
Sethole [26]	2019	Questionnaire	South Africa	N/A	N/A	67.2*	N/A
Parsotam [31]	2020	Questionnaire	United States, Australia, Canada, Ireland, New Zealand, and the United Kingdom	N/A	N/A	N/A	58
Kang [17]	2020	Questionnaire	South Korea	13.3	N/A	N/A	N/A

Tefera [18]	2020	Questionnaire	Africa	37.7	N/A	N/A	N/A
Qureshi [a] [19]	2022	Questionnaire	United States	0–11.1	N/A	N/A	N/A
Lee [20]	2022	Questionnaire	South Korea	44.5*	52*	57.8*	N/A
Antunes-Raposo [21]	2022	Questionnaire	Portugal	57.5	80.4	98.7	N/A
Hayashi [22]	2023	Questionnaire	Japan	60	94	91	N/A

[a] Usage rate before intervention

* Percentage of respondents who reported always wearing a personal dosimeter

N/A: not available

Supplementary Table 2 Definitions of medical specialities referred to in this study

Medical speciality	Subcategories of medical specialities
Cardiology	Cardiological Internal Medicine, Cardiovascular Surgery, Circulatory Surgery, and Vascular Surgery
Gastroenterology	Gastrointestinal Surgery, Gastrointestinal Internal Medicine, and Hepato-Biliary-Pancreatic Internal Medicine
Orthopaedic Surgery	Orthopaedic Surgery, and Spinal Cord Surgery
Neurology	Cerebrovascular Internal Medicine, Neuroendovascular Therapy, Stroke Internal Medicine, Neurological Internal Medicine, and Stroke
Urology	Urology, Dialysis, Kidney Centre Internal Medicine, Nephrological Internal Medicine, and Nephrology-Hypertension Internal Medicine
Radiology	Radiology
Surgery	Surgery, and Breast Surgery
Anaesthesiology	Anaesthesiology, and Pain Clinic
Emergency Medicine	Emergency Medicine, and Intensive Care Unit
Pulmonology	Pulmonology, Respiratory Internal Medicine, and Respiratory Surgery
General Internal Medicine	Internal Medicine, General Clinical Internal Medicine, and Diabetes-Metabolism Internal Medicine
Others	Plastic Surgery, Paediatrics, Gynaecology, Rehabilitation, Residents, Cancer Treatment Centre, and Training Centre
